# Seroprevalence and Risk Factors of Transfusion‐Related Infections Among Blood Donors in Central China During and After the COVID‐19 Pandemic: Evaluation of TTIs in Shiyan

**DOI:** 10.1155/jotm/7023560

**Published:** 2025-12-28

**Authors:** Daiqian Zhu, Danmei Jiao, Fang Tian, Ming Lv, Yao Zhang, Hao Xing, Yufeng Li, Shengnan Wei, Jian Li

**Affiliations:** ^1^ Department of Human Parasitology, School of Basic Medical Science, Hubei University of Medicine, Shiyan, China, hbmu.edu.cn; ^2^ Shiyan Blood Transfusion Center, Laboratory Medical Center, Shiyan, China

**Keywords:** blood donors, COVID-19, hepatitis virus, human immunodeficiency virus, transfusion-transmissible infections, *Treponema pallidum*

## Abstract

**Objective:**

Bloodborne pathogens including hepatitis B virus (HBV), hepatitis C virus (HCV), human immunodeficiency virus (HIV), and *Treponema pallidum* pose a significant menace to transfusion medicine and the public health system. Conducted during the unprecedented global COVID‐19 pandemic, this retrospective analysis (2020–2024) evaluated blood donors from Shiyan, China, aiming to ensure blood safety amidst the challenges posed by the pandemic.

**Methods:**

Cases of transfusion‐transmitted infections (TTIs) were analyzed by using ELISA. Infection rates were quantified as stratified percentages based on sociodemographic characteristics of the study population. Chi‐square trend tests were performed to examine the variation in trends. The chi‐square test was performed to evaluate associations between sociodemographic characteristics (age, gender, and occupation) and the risk of pathogen infection (HBV, HCV, HIV, and *T*. *pallidum*).

**Results:**

Analysis of 231,733 blood samples from 114,593 donors revealed an overall 0.51% seropositivity for TTIs, with prevalence rates of HBV (0.23%), HCV (0.03%), HIV (0.03%), and syphilis (0.22%). During the COVID‐19 pandemic, a declining trend was observed for all infections. The chi‐square test disclosed that HBV and *T*. *pallidum* infection were higher in the other occupation group of women aged 35–60 years, HCV positivity was more pronounced in men in the same group, and HIV infection was mainly concentrated in the group of men aged 25–44 years.

**Conclusions:**

Continuous monitoring of the prevalence of TTIs among blood donors offers valuable information for ensuring blood safety and new directions in the development of blood transfusion systems.

## 1. Introduction

Hemocytes are fundamental to a range of physiological functions, including immunity, oxygen transport, and coagulation. Blood transfusion constitutes a critical method of human clinical treatment and is among the most prevalent therapeutic interventions globally [1]. If blood is contaminated at any point in storing, transporting and clinical application could lead to serious menace to regional or even global health [2]. In developed countries, the rapid improvement of healthcare conditions and the screening for blood donors have led to a sharp decrease in transfusion‐transmitted infections (TTIs), but the infection continues to spread in developing countries, where the TTIs are still transmitted through unscreened blood transfusion and nonsterile injections [3, 4]. Unsafe blood contact increases the risk of patients being exposed to TTIs, including hepatitis B virus (HBV), hepatitis C virus (HCV), human immunodeficiency virus (HIV), and the syphilis‐causing *Treponema pallidum* (*T. pallidum*). The WHO’s report on blood safety and availability displays that 99.8% of blood donations in high‐income countries are screened using basic quality procedures, compared with 99.9% in upper‐middle‐income countries. This compares with 83% in lower‐middle‐income countries and 76% in low‐income countries. The prevalence of TTIs in blood donations is significantly lower in high‐income countries than in low‐ and middle‐income countries. Chinese blood safety has improved significantly through the strengthening management of the blood donation system, the screening for TTIs of all donated blood, the rational use of blood and blood products, and the establishment of well‐organized blood transfusion services. However, TTIs remain a potential threat to blood safety that requires continued attention in the use of blood in China [5].

The results of the fourth national serological survey of hepatitis B in October 2024 revealed that the hepatitis B surface antigen (HBsAg) positivity rate in China has decreased to 5.86%, representing a nearly 20% reduction compared with the previous survey [6]. Notably, the positivity rate among children under 5 years old have dropped to 0.3%, achieving ahead of the WHO Western Pacific Region’s 2025 control target, reducing chronic HBV infection prevalence to below 0.5% in this age group. This progress brings China closer to realize the WHO‐proposed 2030 target of 0.1% HBsAg prevalence rate among children under five years old, and China is expected to reach this goal by 2029 [6]. The number of existing hepatitis C cases continues to decline steadily. The overall prevalence of Acquired Immune Deficiency Syndrome (AIDS) is controlled at a low level and transmission of HIV through blood transfusions and blood products has been effectively blocked, with the mother‐to‐child transmission (MTCT) rate dropping from 7.1% in 2012 to 1.3% in 2023. At the present stage, both MTCT and transmission through injecting drug use have fallen to historic lows in China. In addition, treatment coverage and viral suppression success rates among HIV‐infected person exceed 95% [7–9]. As demonstrated in earlier studies, between January 2015 and December 2019, the seroprevalence rates of HBV, HCV, HIV, and syphilis in the blood donor population in Shiyan, China, are found to be 0.40%, 0.19%, 0.08%, and 0.40%, respectively [10].

The coronavirus disease 2019 (COVID‐19) pandemic, caused by severe acute respiratory syndrome coronavirus 2 (SARS‐CoV‐2), has generated profound global repercussions, severely disrupting core societal functions. Critical public health interventions, including restrictions on population mobility, the implementation of social distancing measures, and widespread disruptions to healthcare delivery systems, have significantly impaired transfusion services worldwide. These disruptions have precipitated critical shortages in the blood supply across numerous regions, jeopardizing essential medical care [11–13]. While the operational challenges and supply deficits faced by blood services are increasingly documented, the specific impact of the pandemic on the risk profile for TTIs within the blood donor population remains inadequately characterized and constitutes a significant knowledge gap. While current reports primarily indicate that respiratory viruses are generally not transmitted via blood transfusion, the lack of definitive evidence elucidating the impact of the COVID‐19 pandemic on TTIs dynamics underscores the necessity and urgency of conducting dedicated epidemiological investigations [14, 15]. Robust seroepidemiological surveys and data analyses focusing specifically on blood donor populations during and in the after‐pandemic period are critically needed. Generating high‐quality evidence is of paramount importance, as it will not only guide the refinement of current risk mitigation strategies but also strengthen the resilience of blood service systems, ultimately ensuring the sustained provision of safe and adequate life‐saving blood supplies for all patients in need. The timeliness of this investigation holds critical significance, as prompt execution is essential to translate empirical findings into actionable policy enhancements and practical improvements.

Consequently, this study aims to evaluate the impact of the COVID‐19 pandemic on the sociodemographic characteristics of blood donors and the seroprevalence of TTIs, specifically HIV, HBV, HCV, and syphilis, among donors in Shiyan City, Hubei Province, located in Central China, from January 2020 to December 2024. Through the continuous monitoring and updating of donor data, it will offer an evidence base for optimizing the management of blood collection and supply systems, ensuring the safety of blood products, and securing blood availability. By analyzing sociodemographic donor characteristics and TTIs trends during this period, we seek to evaluate how the pandemic influenced blood safety and donor profiles. The findings will provide an evidence‐based foundation for enhancing blood collection management, ensuring product safety, and strengthening preparedness for future public health emergencies. Given the extensive impact of the COVID‐19 pandemic on public health infrastructure, this study contributes to safeguarding and enhancing blood safety, supply security, and blood quality during critical periods.

## 2. Methods

### 2.1. Design, Setting, and Subjects Studied

Retrospective analysis of data was performed between January 2020 and December 2024, who donated blood from Shiyan Regional Blood Transfusion Center and Hubei University of Medicine. Volunteers needed to be between the ages of 18 and 60 with body weight ≥ 45 kg, and individuals who met the requirements should complete predonation questionnaires which cover medical history, basic personal information, and sociodemographic characteristics before blood collection. Blood samples were collected in sterile blood collection equipment according to standard venipuncture procedure.

### 2.2. ABO Blood Grouping and Rhesus Typing

ABO and rhesus typing were determined by antigen–antibody agglutination tests which was the general standard reagents, that is, pooled A, B cells (Kinghawk Pharmaceutical Co., Ltd., Beijing, China), anti‐A, anti‐B antisera (Bode Co., Ltd., Changchun, China), and anti‐D antisera (Millipore UK Ltd., Livingston, United Kingdom), according to the kit‐specific instructions to complete typing test [16]. ABO blood group was confirmed when forward and reverse typing results are concordant.

### 2.3. Immunoanalysis of HIV, HBV, HCV, and *T. pallidum*

Donor blood samples were tested by kits which had different screening functions. The ELISA kits (Wantai Biological Pharmacy Enterprise Co., Ltd., Beijing, China; Livzon Diagnostics Inc., Zhuhai, China) were used to detect to HBsAg and antibodies against HCV in serum samples. Meanwhile, serum samples were screened for HIV and *T. pallidum* by ELISA kits (Wantai Biological Pharmacy Enterprise Co., Ltd., Beijing, China; Intec Products Inc., Xiamen, China). All samples were screened two rounds according to the different manufacturers standard test procedure, and any positive results would be disposed of according to the disposal methods mentioned by the Department of Medical Administration National Health Commission (NHC) of China in Technical Operating Procedures for Single‐Donor Plasma Collection Centers (2022). Unqualified samples should be autoclaved and handed over to qualified medical waste disposal units for disposal with proper handover records [17].

### 2.4. Statistical Analysis

Data validation and analysis were performed the Statistical Package for Social Science (SPSS Inc., v. 22.0, USA). The 95% confidence intervals (95% CIs) were established by statistical analysis of ratios after weighting the number of persons tested to report the prevalence of TTIs in the different years and in each subgroup of sociodemographic characteristics considered. Trends about the prevalence of TTIs were analyzed by chi‐square trend test (linear‐by‐linear association). The chi‐square test is used to investigate whether there is a significant association between each TTI and each of the sociodemographic variables. The *p* < 0.05 was considered statistically significant.

## 3. Results

### 3.1. Sociodemographic Characteristics of the Donors

This study involved 114,593 blood donors, recorded between January 2020 and December 2024 (Table 1). Of the total number of blood donors, there were more males (55.83%) than females (44.17%). Of the blood donors grouped by age, the highest percentage was 45–60 years (32.77%), followed by 35–44 years (27.79%), and the smallest percentages were 18–24 years (19.92%) and 25–34 years (19.52%). When categorized by blood group, type O blood was the most prevalent (32.66%), followed by type A (31.10%) and type B (26.92%), the least was AB (9.32%). When classified according to occupation, student constituted the largest percentage of blood donors (20.04%), followed by worker (7.24%) and medical personnel (5.37%). For residents, a significant number (48.04%) did not provide a clear occupational designation.

**Table 1:** Sociodemographic characteristics and seroprevalence of hepatitis B virus (HBV), hepatitis C virus (HCV), human immunodeficiency virus (HIV), and *Treponema pallidum* among blood donors

Characteristics	Number of all blood donors (%)	HBV			HCV			HIV			*T. Pallidum*		
		No. (%, 95% CI)	OR (95% CI)	*p* values	No. (%, 95% CI)	OR (95% CI)	*p* values	No. (%, 95% CI)	OR (95% CI)	*p* values	No. (%, 95% CI)	OR (95% CI)	*p* values
Gender													
Male	63,972 (55.83)	260 (0.40, 0.36–0.45)	1.34 (1.13–1.59)	0.001	41 (0.06, 0.04–0.08)	0.83 (0.51–1.35)	0.458	40 (0.06, 0.04–0.08)	0.73 (0.43–1.21)	0.220	222 (0.35, 0.30–0.39)	1.67 (1.40–1.99)	< 0.001
Female	50,621 (44.17)	275 (0.54, 0.48–0.60)	1	N/A	27 (0.05, 0.03–0.07)	1	N/A	23 (0.05, 0.03–0.06)	1	N/A	293 (0.58, 0.51–0.64)	1	N/A
Age group (years)													
18–24	22,828 (19.92)	63 (0.28, 0.21–0.34)	1.77 (1.33–2.36)	< 0.001	15 (0.07, 0.03–0.10)	0.97 (0.51–1.85)	0.933	16 (0.07, 0.04–0.10)	0.30 (0.13–0.71)	0.004	49 (0.21, 0.15–0.27)	2.23 (1.62–3.06)	< 0.001
25–34	22,307 (19.52)	122 (0.54, 0.45–0.64)	0.89 (0.71–1.12)	0.334	11 (0.05, 0.02–0.08)	1.30 (0.64–2.65)	0.470	20 (0.09, 0.05–0.13)	0.24 (0.10–0.54)	< 0.001	73 (0.33, 0.25–0.40)	1.46 (1.11–1.92)	0.006
35–44	31,846 (27.79)	167 (0.52,0.44–0.60)	0.93 (0.75–1.15)	0.493	18 (0.06, 0.03–0.08)	1.13 (0.61–2.08)	0.693	19 (0.06, 0.03–0.09)	0.34 (0.15–0.77)	0.007	214 (0.67, 0.58–0.76)	0.71 (0.58–0.86)	0.001
45–60	37,549 (32.77)	183 (0.49, 0.41–0.56)	1	N/A	24 (0.06, 0.04–0.09)	1	N/A	8 (0.02, 0.01–0.04)	1	N/A	179 (0.47, 0.41–0.54)	1	N/A
Occupation													
Worker	8291 (7.24)	47 (0.56, 0.40–0.72)	1.02 (0.75–1.38)	0.920	4 (0.05, 0.00–0.10)	1.47 (0.52–4.11)	0.461	2 (0.02, −0.01–0.06)	2.26 (0.54–9.46)	0.251	49 (0.59, 0.42–0.75)	0.98 (0.73–1.33)	0.897
Farmer	5085 (4.44)	30 (0.59, 0.38–0.80)	0.98 (0.67–1.42)	0.899	4 (0.08, 0.00–0.16)	0.90 (0.32–2.52)	0.842	3 (0.06, −0.01–0.13)	0.92 (0.28–3.02)	0.896	61 (1.19, 0,89–1.48)	0.48 (0.36–0.63)	< 0.001
Student	22,959 (20.04)	45 (0.20, 0.14–0.25)	2.95 (2.16–4.03)	< 0.001	14 (0.06, 0.03–0.09)	1.16 (0.63–2.14)	0.630	8 (0.03, 0.01–0.06)	1.56 (0.72–3.41)	0.257	37 (0.16, 0.1–0.21)	3.61 (2.57–5.08)	< 0.001
Merchant	3175 (2.77)	22 (0.69, 0.40–0.97)	0.83 (0.54–1.28)	0.399	2 (0.06, −0.02–0.15)	1.12 (0.27–4.66)	0.871	4 (0.13, 0.00–0.25)	0.43 (0.15–1.23)	0.105	14 (0.44,0.21–0.67)	1.32 (0.77–2.25)	0.314
Medical personnel	6148 (5.37)	34 (0.55, 0.37–0.73)	1.04 (0.73–1.48)	0.822	2 (0.03, −0.01–0.08)	2.18 (0.53–9.02)	0.271	5 (0.08, 0.01–0.15)	0.67 (0.26–1.73)	0.404	11 (0.18, 0.07–0.28)	3.25 (1.78–5.93)	< 0.001
Teacher	3365 (2.94)	13 (0.38, 0.18–0.59)	1.49 (0.86–2.60)	0.155	0 (0.00, 0.00–0.00)	2.45 (0.34–17.81)	0.731	5 (0.15, 0.02–0.28)	0.37 (0.14–0.94)	0.030	5 (0.15, 0.02–0.28)	3.92 (1.62–9.48)	0.001
Govt. employee	5605 (4.89)	11 (0.20, 0.08–0.31)	2.95 (1.61–5.38)	< 0.001	2 (0.04, −0.01–0.09)	1.99 (0.48–8.23)	0.335	4 (0.07, 0.00–0.14)	0.76 (0.27–2.17)	0.611	7 (0.12, 0.0300.22)	4.66 (2.20–9.86)	< 0.001
Soldier	584 (0.51)	0 (0.00, 0.00–0.00)	3.40 (0.48–24.24)	0.272	0 (0.00, 0.00–0.00)	0.43 (0.06–3.10)	0.352	0 (0.00, 0.00–0.00)	0.33 (0.04–2.42)	0.287	0 (0.00, 0.00–0.00)	3.42 (0.48–24.40)	0.272
Technology professional	4329 (3.78)	16 (0.37, 0.19–0.55)	1.56 (0.94–2.58)	0.080	1 (0.02, −0.02–0.07)	3.07 (0.42–22.34)	0.244	2 (0.05, −0.02–0.11)	1.18 (0.28–4.94)	0.821	12 (0.28, 0.12–0.43)	2.10 (1.18–3.73)	0.010
Other	55,052 (48.04)	317 (0.58, 0.51–0.64)	1	N/A	39 (0.07,0.05–0.09)	1	N/A	30 (0.05, 0.04–0.07)	1	N/A	319 (0.58, 0.51–0.64)	1	N/A

*Note:* No., N/A, OR, and 95% CI represent number, no data, odds ratios, and 95% confidence interval, respectively.

### 3.2. Seropositivity and Risk Factors Evaluation of Transfusion‐Related Infections

**Figure 1:**
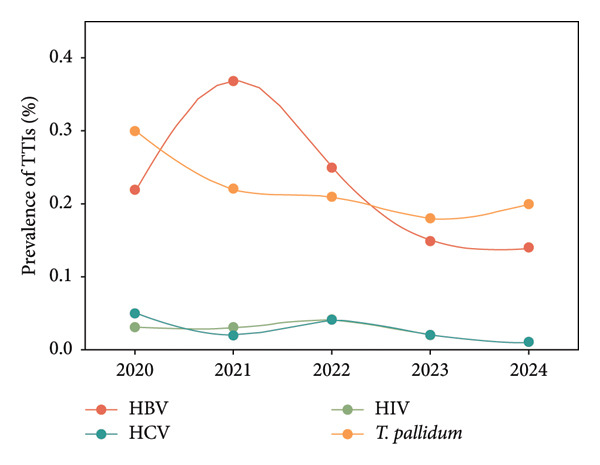
Prevalence of transfusion‐transmitted infections (TTIs) in 2020–2024. For TTIs, they include hepatitis B virus (HBV), hepatitis C virus (HCV), human immunodeficiency virus (HIV), and *Treponema pallidum*.

During the five years of the study, 231,733 blood samples have been tested, of which 1181 (0.51%, 95% CI: 0.48–0.54) were positive for TTIs (Table 2). The prevalence of HBV, HCV, HIV, and syphilis among all blood samples was 0.23%, 0.03%, 0.03%, and 0.22%, respectively. It was observed that the prevalence of HBV in all blood samples over the five‐year period reached a pinnacle of 0.37% (95% CI: 0.32–0.42) in 2021 from 0.22% (95% CI: 0.18–0.26) in 2020. Thereafter, a yearly decline was observed, reaching 0.14% (95% CI: 0.11–0.18) in 2024. For HCV infection, it was decreased from 0.05% (95% CI: 0.03–0.07) in 2020 to 0.02% (95% CI: 0.01–0.04) in 2021, increased to 0.04% (95% CI: 0.02–0.05) in 2022, and reached a low of 0.01% (95% CI: 0.00–0.03) in 2024. The HIV prevalence remained at 0.03% (95% CI: 0.01–0.04) and 0.03% (95% CI: 0.01–0.05) for the first two years (2020‐2021), then increased to 0.04% (95% CI: 0.02–0.06) in 2023, and fell to a low of 0.01% (95% CI: 0.00–0.03) in 2024. The prevalence of syphilis decreased from 0.30% (95% CI: 0.25–0.35) in 2020 to 0.20% (95% CI: 0.16–0.24) in 2024 (Figure 1).

**Table 2:** Seropositivity of hepatitis B virus (HBV), hepatitis C virus (HCV), human immunodeficiency virus (HIV), and *Treponema pallidum* among blood donors in 2020–2024.

Year	Total screened No.	HBV No. (%, 95% CI)	HCV No. (%, 95% CI)	HIV No. (%, 95% CI)	*T. pallidum* No. (%, 95% CI)
2020	46,085	102 (0.22, 0.18–0.26)	24 (0.05, 0.03–0.07)	13 (0.03, 0.01–0.04)	138 (0.30, 0.25–0.35)
2021	49,999	185 (0.37, 0.32–0.42)	12 (0.02, 0.01–0.04)	15 (0.03, 0.01–0.05)	108 (0.22, 0.18–0.26)
2022	46,761	118 (0.25, 0.21–0.30)	17 (0.04, 0.02–0.05)	18 (0.04, 0.02–0.06)	99 (0.21, 0.17–0.25)
2023	47,875	71 (0.15, 0.11–0.18)	9 (0.02, 0.01–0.03)	11 (0.02, 0.01–0.04)	88 (0.18, 0.15–0.22)
2024	41,013	59 (0.14, 0.11–0.18)	6 (0.01, 0.00–0.03)	6 (0.01, 0.00–0.03)	82 (0.20, 0.16–0.24)
*p* values	—	0.000	0.002	0.183	0.001
Total	231,733	535 (0.23, 0.21–0.25)	68 (0.03, 0.02–0.04)	63 (0.03, 0.02–0.03)	515 (0.22, 0.20–0.24)

*Note:* CI, confidence interval.

A chi‐square test analysis was used to systematically assess the risk factors for TTIs in the blood donor population during the period 2020–2024 (Table 1). The data demonstrated significant differences in the gender distribution of HBV (OR = 1.34, 95% CI: 1.13–1.59, and *p* = 0.001) and syphilis (OR = 1.67, 95% CI: 1.40–1.99, and *p* < 0.0001) infections, with male blood donors have lower seropositivity rates than females significantly. The gender differences in HCV (OR = 0.83, 95% CI: 0.51–1.35, and *p* = 0.458) and HIV (OR = 0.73, 95% CI: 0.43–1.21, and *p* = 0.220) infection were not statistically significant.

The groups according to age indicate that the prevalence of HBV (OR = 1.77, 95% CI: 1.33–2.36, and *p* < 0.0001) and syphilis (OR = 2.23, 95% CI: 1.62–3.06, and *p* < 0.0001) is significantly different between 18–24 years and 45–60 years, with the younger group exhibiting a lower prevalence of infections. The prevalence of HCV was not different in the younger donors (OR = 0.97, 95% CI: 0.51–1.85, and *p* = 0.933) and the prevalence of all ages were maintained at a low level. The prevalence of HIV among 18–24 years was significantly higher than 45–60 years (OR = 0.30, 95% CI: 0.13–0.71, and *p* = 0.004). A significant difference was observed between the 45–60‐year age group and the 25–34‐year age group (OR = 0.24, 95% CI: 0.10‐0.54, *p* < 0.001). A significant difference was also found between the 45–60‐year and the 35–44‐year age groups (OR = 0.34, 95% CI: 0.15‐0.77, *p* = 0.007).

Analysis of the positivity rate for different occupations showed that merchant exhibited the highest HBV positivity rate (0.69%), farmer exhibited the highest HCV positivity rate (0.08%) and the highest syphilis positivity rate (1.19%), and teacher exhibited the highest HIV positivity rate (0.15%). A chi‐square test analysis exposed that there was a significant difference in the risk of HBV infection between student (OR = 2.95, 95% CI: 2.16–4.03, and *p* < 0.001) and Govt. employee (OR = 2.95, 95% CI: 1.16–5.38, and *p* < 0.0001). There were no significant differences in HCV infection rates in all occupations (*p* > 0.05). Teacher with the highest HIV prevalence (0.15%, 95% CI: 0.02–0.28) exhibited a significant negative correlation (OR = 0.37, 95% CI: 0.14–0.94, and *p* = 0.030), whereas worker (OR = 2.26, 95% CI: 0.54–9.46, and *p* = 0.251), student (OR = 1.56, 95% CI: 0.72–3.41, and *p* = 0.257), and technology professional (OR = 1.18, 95% CI: 0.28–4.94, and *p* = 0.821) showed a trend of positive correlation (Table 1).

### 3.3. Effect of COVID‐19 on the Prevalence of TTIs

In this study, to monitor the impact of COVID‐19 on the prevalence of TTIs among blood donors in Shiyan City, the collected dataset was stratified into two distinct periods: the during‐COVID‐19 period (2020–2022) and the after‐COVID‐19 period (2023–2024). The corresponding blood sample sizes were 142,845 and 88,888, respectively.

**Figure 2:**
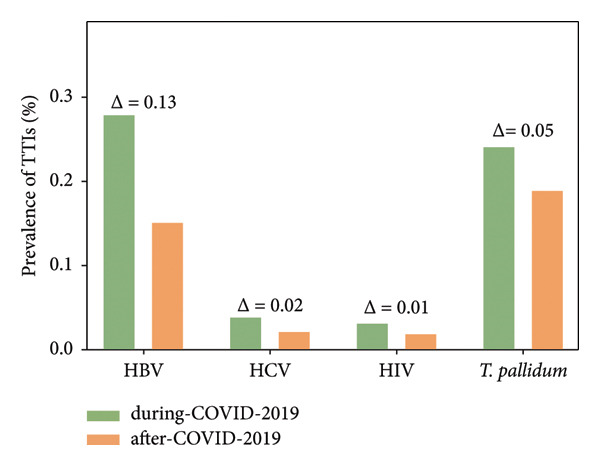
Comparative TTIs prevalence: During versus After COVID‐19. The prevalence of HBV, HCV, HIV, and *Treponema pallidum* comparing between COVID‐19 pandemic (2020–2022) and after COVID‐19 (2023‐2024), and their prevalence difference (Δ).

To visually demonstrate changes in the prevalence of TTIs between the during‐COVID‐19 and after‐COVID‐19 periods, it presents statistical results in percentages (%). Data indicate that compared with the during‐COVID‐19 period (2020–2022), the after‐COVID‐19 period (2023–2024) showed a significant reduction of 0.13% in HBV prevalence (from 0.28% to 0.15%), a decrease of 0.02% in HCV prevalence (from 0.04% to 0.02%), a decline of 0.01% in HIV prevalence (from 0.03% to 0.02%), and a drop of 0.05% in syphilis prevalence (from 0.24% to 0.19%) (Figure 2).

Compared with the period during COVID‐19, the HBV prevalence among blood donors demonstrated a marked decline across all sociodemographic subgroups following the conclusion of the COVID‐19 pandemic. Among HBV‐positive cases, the magnitude of the prevalence reduction varied by population characteristics, where female donors (55.15%), young donors (18–24 years, 89.10%), and student donors (84.32%) exhibited the most substantial decreases.

Although the number of HCV‐positive cases was limited, the overall HCV prevalence still demonstrated a declining trend. In terms of percentage changes, substantial decreases were observed among male donors (61.04%), young donors (24–34 years, 83.93%), and student donors (56.17%). Merchant, medical personnel, Govt. employee, and technology professional exhibited a 100% reduction in HCV prevalence. In contrast, HCV prevalence among industrial worker donors increased significantly by 60.70%.

**Figure 3:**
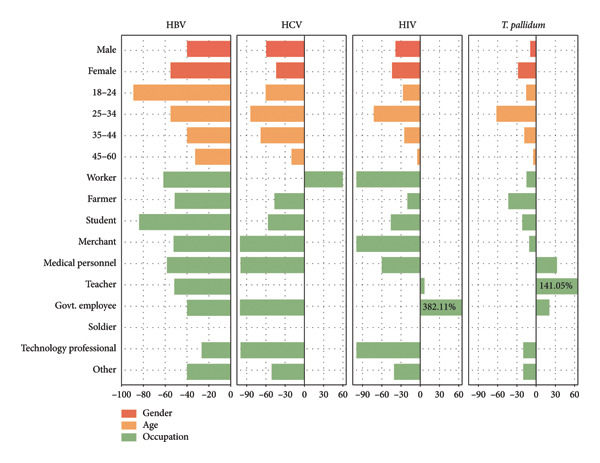
Comparative TTIs seroprevalence by sociodemographic characteristics during versus after COVID‐19. The prevalence difference of TTIs comparing between COVID‐19 pandemic (2020–2022) and after COVID‐19 (2023–2024) by Sociodemographic characteristics of the blood donors. Numerals in parentheses represent actual values surpassing the axis limit.

Among the studied TTIs, HIV prevalence exhibited the minimal fluctuation. A 39.04% reduction was observed in male donors, while female donors showed a 43.28% decline. The most substantial decrease (71.64%) occurred in the 25–34‐year age group. Prevalence changes varied significantly across occupational categories. Medical personnel demonstrated a 59.82% reduction, whereas industrial worker, merchant, and technology professional achieved complete elimination (100%). Conversely, teacher exhibited a 7.13% increase, with Govt. employee donors displaying a dramatic surge of 382.11%. Syphilis infection burden was comparable to HBV, with male prevalence decreasing by 9.46% and female prevalence declining by 28.75%. The most pronounced decline (61.87%) occurred among 25–34‐year‐old donors. Farmer demonstrated a significant 42.86% reduction in syphilis prevalence. Conversely, medical personnel (33.92%), teacher (141.05%), and Govt. employee (20.53%) exhibited marked upward trends. Notably, soldier showed complete absence of TTIs seropositivity across both datasets (Figure 3).

## 4. Discussion

This study examined the trends of TTIs among blood donors in Shiyan, Central China, from 2020 to 2024, a period encompassing the COVID‐19 pandemic and its aftermath. The overall seroprevalence of TTIs during this interval was 0.51%, which is notably lower than the rates reported in the previous five‐year periods (1.07% for 2015–2019 and 1.35% for 2010–2014) [10, 18]. This continuing decline likely reflects enhanced public health measures, donor screening improvements, and pandemic‐related changes in behavioral. These findings underscore the importance of sustained surveillance and adaptable public health strategies to maintain blood safety amid evolving epidemiological contexts.

According to the WHO 2023 data, HBV remains highly prevalent in lower middle‐income (2.81%) and low‐income countries (1.70%) [5]. A global meta‐analysis of 3740 studies showed a substantial decline in HBsAg seroprevalence, from 9.6% (from 1973 to 1984) to 3.0% in 2021 [19]. In China, HBV poses the highest disease burden among infectious diseases. This study recorded an HBV prevalence of 0.23% in blood donors; its prevalence among blood donors has steadily decreased from 0.51% (2010–2014) to 0.40% (2015–2019), reflecting broad improvements in prevention and control [20]. This downward trend can be largely attributed to effective public health strategies, including widespread vaccination and health education campaigns. Notably, prevalence varies across occupations, with higher rates observed among farmers and merchants. The decline among farmers may reflect improved rural health initiatives, while the slight increase among merchants could be associated with occupational exposure and mobility. Moving forward, reducing MTCT remains essential for improving long‐term outcomes [21]. Although China has made progress through perinatal interventions [22], new challenges such as population aging require continued adaptation of HBV management strategies to align with the WHO elimination targets [23].

A notable finding was the significant decline in HCV prevalence to 0.03% in 2020–2024, reaching the lowest level across all demographic groups in 15 years. This reduction may reflect enhanced screening and public health measures during the pandemic. As we all know, the epidemiology of primary liver cancer in adults is continuously development, driven by the rising prevalence of metabolic diseases, has led to liver cancer being one of the leading causes of global mortality [24]. Despite regional heterogeneity in HCV genotypes in China [25], and the absence of an effective vaccine, advances in antigen design offer promising directions [26]. However, high infection rates among people who inject drugs highlight the need for targeted interventions. Strengthening awareness and curbing high‐risk behaviors are essential to meet the WHO HCV elimination goals by 2030 [27]. The China’s efforts about ongoing research and policy adjustments are crucial for facing the evolving challenges in HCV prevention and control.

HIV prevalence in Shiyan declined to 0.03%, reflecting broader prevention efforts. However, the infection burden has shifted among age groups, with the highest rate currently observed among those aged 25–34 (0.09%). Students previously showed the highest occupational infection rate (0.12%) and this has a lot to do with casual sex and lack of sex education [28]. Recent data indicate elevated prevalence among teachers (0.15%) and merchants (0.13%) in the present study, suggesting both shifting risk profiles and possible effects of targeted interventions. HIV rejuvenation remains a concern, particularly among youth [29]. To address these challenges, it is imperative to strengthen the implementation of comprehensive sexual education programs. Schools should serve as the primary platform for disseminating foundational knowledge about sexual health and educate the public about transmission routes and the severe consequences of HIV infection at the same time.

Syphilis prevalence in Shiyan has consistently declined to 0.22%. Women showed higher infection rates than men throughout this period. This reduction can be largely attributed to robust public health interventions, including nationwide free antenatal syphilis screening, a cost‐effective strategy that enhances detection, prevents vertical transmission, and facilitates integrated sexually transmitted disease (STD) and HIV control [30, 31]. Furthermore, this decline was closely associated with the comprehensive strengthening of containment measures during the COVID‐19 pandemic. Nonpharmaceutical interventions, such as social distancing, mask‐wearing, and restrictions on gatherings, effectively reduced opportunities for pathogen transmission via respiratory, contact, and blood/body fluid routes, thereby indirectly mitigating the risk of infection through established TTI transmission pathways.

The COVID‐19 pandemic significantly reduced the transmission of TTIs due to restricted social interactions. This study observed a pronounced decline in HBV prevalence (13.73%), with notable decreases among students, female, and young/middle‐aged donors (18–44 years). However, certain occupational groups showed increased seropositivity, possibly due to cross‐reactivity between SARS‐CoV‐2 and HIV, highlighting the need for confirmatory testing and exposure history reviews [32, 33]. Although COVID‐19 vaccination and infection status may influence serological outcomes, these variables were not included due to data limitations. Moving forward, we recommend establishing a resilient blood inventory buffer to mitigate supply disruptions and enhancing public education to maintain a stable donor pool. These measures are critical for addressing future public health emergencies affecting blood supply.

While existing laboratory techniques are efficient and sensitive, the risk from high‐risk donors remains non‐negligible. To enhance blood safety, we recommend modernizing testing assays, incorporating parasitic screening for donors with relevant exposure history, and strengthening donor follow‐up and reporting protocols. Furthermore, increasing public engagement and shortening statistical intervals can improve data timeliness. These measures align with national strategies to reduce infections and mortality while sustaining low prevalence of bloodborne diseases. The COVID‐19 pandemic underscored the need for robust blood inventory buffers and responsive donor management systems to address both sudden shortages and emerging serological challenges.

## 5. Conclusions

This study analyzes the five‐year seroprevalence of TTIs among blood donors in Central China, using Shiyan City as a representative setting, from 2020 to 2024. A notable decline in TTI rates was observed during and after the COVID‐19 pandemic. However, persistent risks due to China’s large population underscore the need for enhanced public health education, optimized screening, and WHO‐guided quality assurance to ensure transfusion safety.

**Nomenclature**
HBV: Hepatitis B virus
HCV: Hepatitis C virus
HIV: Human immunodeficiency virus
TTIs: Transfusion‐transmitted infections
AIDS: Acquired Immune Deficiency Syndrome
MTCT: Mother‐to‐child transmission
CI: Confidence intervals
STD: Sexually transmitted disease

## Ethics Statement

The current study was approved by the Ethics Committees of Hubei University of Medicine (2018‐TH‐003 and 2021‐TH‐080) and the Shiyan Blood Transfusion Center Ethics Committee (SBTCEC‐01). Because of the limitations of this retrospective analysis, however, informed consent was not obtained from the individuals studied.

## Consent

Please see the Ethics Statement.

## Disclosure

All the authors read and approved the final manuscript.

## Conflicts of Interest

The authors declare no conflicts of interest.

## Author Contributions

J.L. and S.W. conceived and designed the study and participated in its design and coordination. D.Z., D.J., F.T., M.L., Y.Z., H.X., and Y.L. collected the data and processed data. D.Z. wrote the manuscript, and JL revised the manuscript.

## Funding

This study was supported by the Principle Investigator Program of Hubei University of Medicine (Grant number HBMUPI202101) and the Foundation of Health Commission of Hubei Province (Grant number WJ2019H334).

## Data Availability

The datasets analyzed in this study are available from the corresponding authors upon reasonable request.
